# Insect proteins and peptides: preparation, bioactivities, and applications

**DOI:** 10.1007/s44463-025-00013-0

**Published:** 2026-02-11

**Authors:** Lingke Wang, Jiayou Li, Yixin Chen, Yang Zhang, Ruyuan Zhang

**Affiliations:** College of Biological and Chemical Engineering, Jiaxing University, Jiaxing, 314001 China

**Keywords:** Insect, Proteins, Peptides, Bioactivities, Applications

## Abstract

The surge in global population has driven a dramatic increase in protein demand, and insects have emerged as a promising sustainable high-quality protein resource due to their advantages of high reproductive rates, low production costs, high protein content, and balanced nutritional profiles. This review systematically compares the traditional and innovative extraction methods for insect proteins and peptides, and explores their functional characteristics. Moreover, this review summarizes the multiple applications of insect proteins and peptides in food, feed, and pharmaceutical industry, and provides a forward-looking perspective on the challenges that impede their broader implementation, and concrete recommendations are proposed to facilitate the industrial advancement of insect-derived bioproducts and foster their integration into mainstream consumer markets.

## Introduction

As the global population continues to expand, there emerges an imperative to explore new sustainable protein sources (Queiroz et al., 2023). Global protein demand is poised to double by 2050 relative to 2010 baseline levels, a change propelled by both population growth and shifts in dietary preferences (Smetana et al., 2015). Among the innovative solutions being investigated, edible insects present a particularly promising alternative protein source, attributable to their favorable nutritional profile, bioactive potential, and superior environmental sustainability metrics when contrasted with traditional livestock production (Traynor et al., 2024).

The class insecta constitutes a significant portion of global biodiversity, encompassing over one million described species, which account for 60% of all documented eukaryotes. Entomological surveys have cataloged approximately 3,650 edible species across 21 orders, with the most nutritionally significant taxa including Coleoptera (31%), Lepidoptera (18%), and Hymenoptera (14%) (Smetana et al., 2016). Current estimations indicate that 80 countries across regions including Asia (932 species), North America (529 species), Africa (464 species), and South America (300 species), 2,205 insect species have already been incorporated as high-quality protein sources into the diets of at least two billion people (Omuse et al., 2024). Consumers in Africa, Asia, and Latin America have a higher acceptance of insect food than those in Europe and North America. For example, Latin American consumers have a 19% higher willingness to try insect food than European consumers (Abro et al., 2025). This phenomenon may be closely linked to underlying cultural disparities. Brazil’s rich entomophagy culture dates back to the 16th century, when during the early European colonial period, indigenous communities had already been consuming diverse insect species as part of their subsistence practices. This tradition has become deeply ingrained in Brazilian culinary heritage. In contrast, across urban societies in European countries, insects are frequently associated with unsanitary environments, thereby creating significant psychological barriers to their acceptance as food.

Previous studies have shown that the edible insect market has grown from USD 400 million in 2018 to USD 1.23 billion in 2023, with a global CAGR of 25, further projections indicate a potential valuation of USD 8 billion by 2030, highlighting its growing commercial and dietary importance (Evanson et al., 2024).

The protein content of insects exhibits substantial variation across species, ranging from 50% to 80% by dry weight (Huseynli et al., 2023), which significantly surpasses conventional sources such as beef (26 ± 3%) and soy (38 ± 2%) (Valentina et al., 2022). In comparison to traditional protein sources, insect protein demonstrates a more balanced nutritional profile (Renske et al., 2017) and higher degree of absorption rate (Igual et al., 2021). It contains over 20 essential amino acids required for human health, including lysine, phenylalanine, and valine—amino acids that the human body cannot synthesize endogenously (Gravel, 2020). Notably, concentrations of lysine (4.3% w/w) and methionine (2.1% w/w) in insect protein exceed those found in cereal proteins by 105% and 89%, respectively (Lívya et al., 2024).

In addition, insect protein has been identified as a precursor for the synthesis of bioactive compounds, which encompass antimicrobial peptides (AMPs), antioxidant peptides, and antihypertensive peptides (Rivero-Pino et al., 2024). The functional properties of insect proteins elevate their status as valuable constituents in the realm of nutraceutical development, thereby reinforcing their applicability in the formulation of functional foods (Sharma et al., 2024) and nutrition supplements (Liang et al., 2024).

Table 1 summarizes the macronutrient profiles of selected dried insect species. Notably, *Tenebrio molitor* (yellow mealworms) and *Bombyx mori* (silkworm pupae) exhibit the highest crude protein contents, quantified at 50.2% and 48.7%, respectively. Conversely, *Zophobas morio* (super mealworms) are characterized by the most elevated crude fat content at 41.71%. Furthermore, *Hermetia illucens* larvae (black soldier fly larvae) manifest a significantly higher crude ash content of 14.6% relative to other species, a factor that may be attributed to their detritivorous feeding habits and unique digestive physiology (Salam et al., 2024). These compositional variations are pivotal when assessing insect species as viable protein sources, as they have substantive implications for both nutritional profiles and industrial applications. For example, *Tenebrio molitor* and *Bombyx mori* are deemed optimal protein sources, while *Zophobas morio* are favored for formulations that require a higher lipid content. Additionally, *Bombyx mori* are noted for their comprehensive amino acid profile, particularly their richness in leucine and isoleucine (Jung, 2021; Song et al., 2023).

**Table 1:** Basic nutrient content of insect-dried bodies (g/100 g dry weight)

Common Name	Housefly larvae	Yellow mealworm	Super mealworm	Black soldier fly larvae	Silkworm pupae
Scientific Name	*Musca domestica*	*Tenebrio molitor*	*Zophobas morio*	*Hermetia illucens*	*Bombyx mori*
Crude protein	45.62	50.20	45.08	42.10	48.70
Crude fat	30.10	28.96	41.71	34.80	30.10
Crude ash	5.97	3.54	2.64	14.60	8.60
Lysine	3.19	2.60	2.38	3.40	3.65
Methionine	0.97	2.20	0.43	0.90	2.24
Threonine	1.78	1.76	1.58	0.60	2.62
Phenylalanine	2.71	1.89	1.70	2.20	2.48
Valine	2.11	3.04	2.69	3.40	2.73
Isoleucine	1.39	1.83	1.63	2.00	2.78
Leucine	2.90	3.64	3.02	3.50	4.04
Arginine	2.31	2.47	2.20	2.20	1.36
Histidine	1.16	1.41	1.20	2.11	1.22
Alanine	2.56	3.40	3.02	3.70	5.50
Tyrosine	2.05	0.31	2.97	2.50	5.40
Aspartic acid	3.97	3.38	3.16	4.60	10.90
Glutamic acid	6.29	4.92	4.50	3.80	14.90
Proline	1.75	3.09	1.93	3.30	4.00
Glycine	1.73	2.31	1.90	2.90	4.60
Serine	1.71	1.82	1.65	0.10	4.70
Reference	Dörper et al. (2021); Hawkey et al.(2020); Jantzen et al. (2020); Jung, (2021); Oonincx et al.(2021); Orkusz (2021); Rumpold et al., (2013); Sergiy et al. (2019); Sharma et al. (2024); Song et al. (2023)				

From a policy perspective, the Food and Agriculture Organization of the United Nations (FAO) has emphasized its significance early in 2013 that edible insects are an underutilized resource with the potential to meet current and future global nutritional needs (Stull & Patz, 2020). Europe has taken the lead in supporting insect-based proteins, having classified insects as “novel foods” since 2018. This classification has legalized their use as ingredients and established strict safety standards. In recent years, countries like South Korea and Malaysia have officially recognized numerous insect species, including *Tenebrio molitor*, *Bombyx mori*, and *Zophobas morio*, as edible resource. In addition, China, Japan, Canada, and Australia have also introduced corresponding policies to promote the development of local insect protein industry (Gasco et al., 2020).

**Fig. 1:**
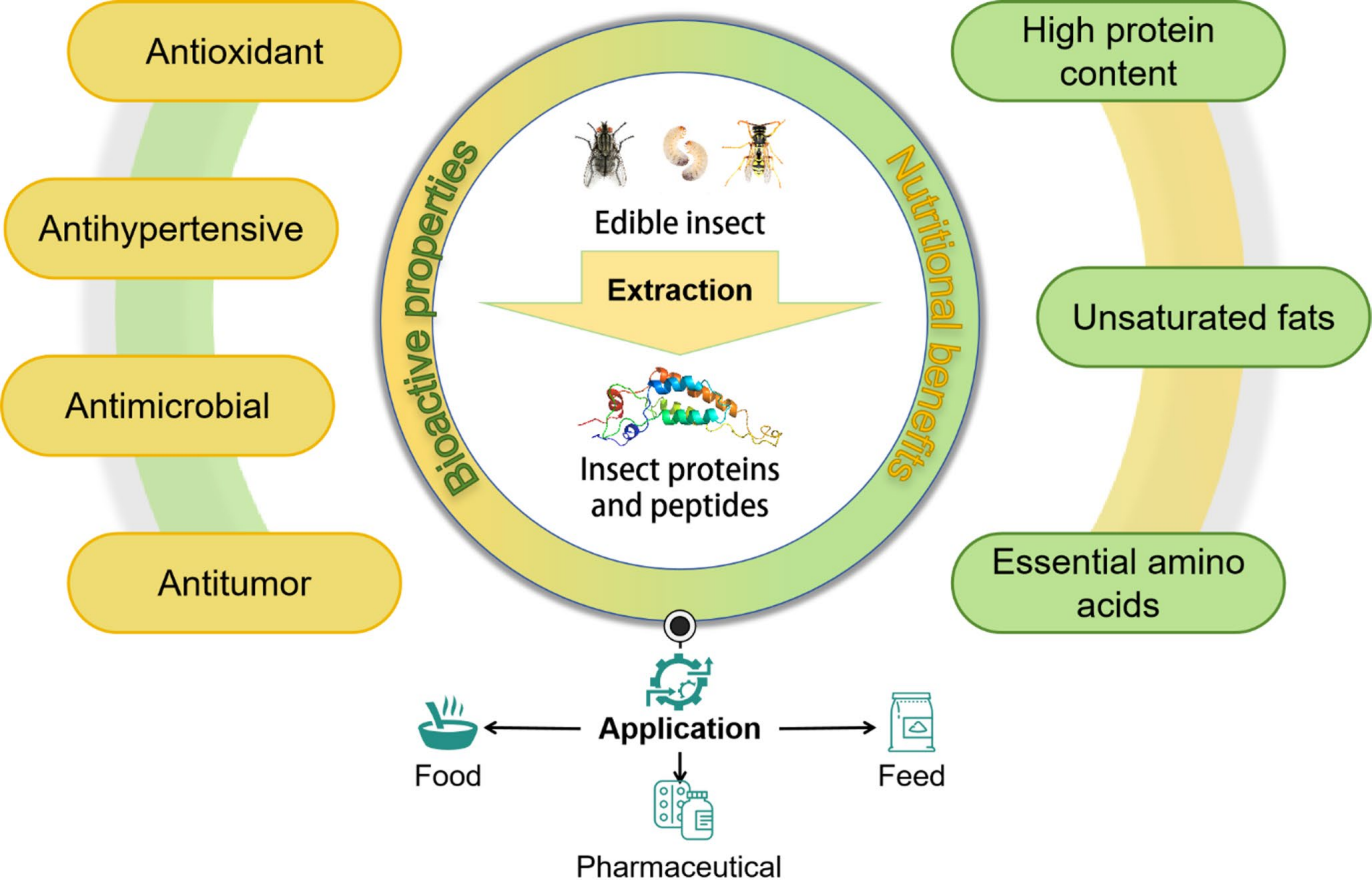
Bioactive properties and nutritional benefits of insect proteins and peptides

Although many scholars have studied the nutritional value and functional characteristics of insect proteins and peptides, proving the feasibility of insects as a source of proteins and peptides, their market penetration in the food and feed industry is still limited. Therefore, this review focuses on the current situation of the insect protein industry, aiming to promote the integration of insect protein into the mainstream consumer market. The nutritional compositions of common insects as protein sources (e.g., *Tenebrio molitor*,* Zophobas morio*,* Hermetia illucens*,* Bombyx mori*) is profiled. This review systematically compares the advantages and disadvantages of traditional (alkali-based, salt-based, protease-based, and Tris-HCl buffer techniques) and innovative extraction methods (supercritical CO_2_ extraction, enzyme-assisted extraction, ultrasonic or microwave-assisted extraction, and dry fractionation technology), with a focus on economic feasibility, yield, and application scope; further delves into the bioactive components of insect-derived materials, including AMPs, C-type lectins (CTLs), polyphenol oxidase (PPO), and fibrinolytic active proteins, analyzing their functional properties and potential applications; explores the prospective uses of insect proteins and peptides in food, feed, and pharmaceutical industries, while dissecting the multifaceted challenges they face—low consumer acceptance, economic competitiveness, safety risk uncertainties, and regulatory restrictions, and concrete recommendations are proposed to facilitate the industrial advancement of insect-derived bioproducts and foster their integration into mainstream consumer markets (Fig. 1).

## Preparation methods of insect proteins and peptides

**Fig. 2:**
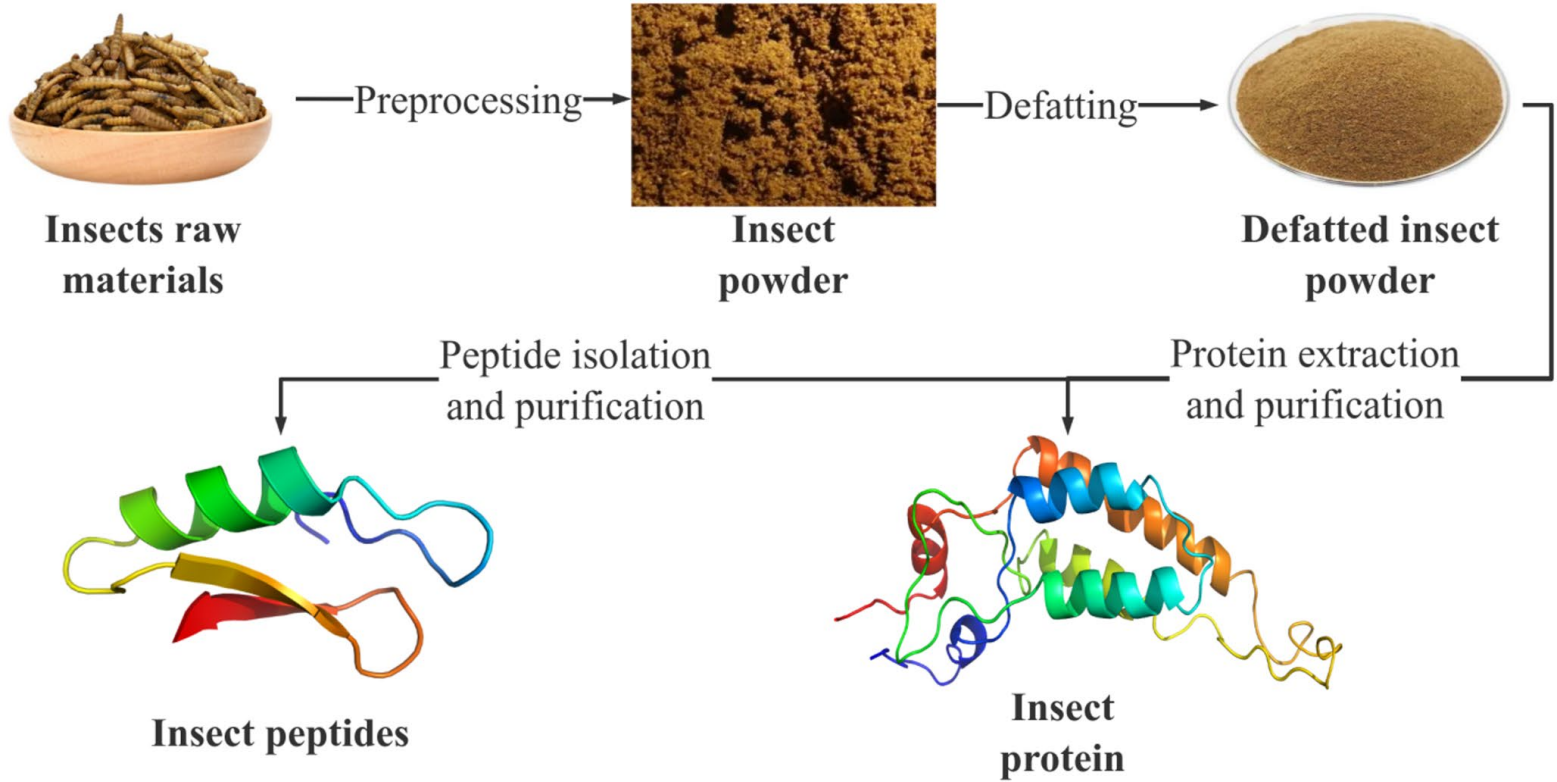
Preparation steps of insect proteins and peptides

The preparation of insect proteins and peptides entails a series of intricate unit operations, as illustrated in Fig. 2. These operations include comminution, drying, disinfection, sterilization, oil removal, filtration, concentration, separation, purification, and hydrolysis. Among these processes, critical operations such as oil removal, protein fractionation, and peptide isolation are governed by specific process parameters, including temperature, pH, and enzyme-substrate ratios (Ma et al., 2023).

### Oil removal

Given the substantial lipid content of insects, which range from 20% to 30% of dry weight, prior lipid extraction is essential to mitigate undesirable interference with downstream protein isolation processes (Sergiy et al., 2019). Table 2 provides a comparative overview of commonly employed lipid extraction methods. Among the various techniques, organic solvent extraction is the most prevalent approach for defatting. Non-polar solvents, such as n-hexane, are frequently utilized for oil removal from several insect species including *Bemisia tabaci*,* Tenebrio molitor*,* Zophobas morio*,* Liriomyza huidobrensis*,* Musca domestica*,* and Chilo suppressalis* (Mittal et al., 2024). As an alternative, petroleum ether is used for lipid extraction from *Bemisia tabaci* and *Bombyx mori* pupae. Polar solvents such as ethanol and isopropanol have also been employed in defatting *Tenebrio molitor* larvae (Xue et al., 2016). However, the utilization of these polar solvents may lead to partial solubilization of alcohol-soluble proteins, consequently resulting in protein loss during processing. Moreover, the incorporation of organic solvents raises both safety and environmental concerns (Soron et al., 2021). Although ethanol is regarded as the safest option among these solvents, it demonstrates marginally lower extraction efficiency when compared to its non-polar counterparts (Song et al., 2015).

**Table 2:** Advantages and disadvantages of common oil extraction methods

Oil Extraction Methods	Advantages	Disadvantages
Organic solvent extraction	Fast extraction speed, simple operation, suitable for the extraction of most organic compounds	Volatile solvents are flammable and evaporate easily; safety precautions are necessary during processing
Enzyme-assisted water extraction	Low operating temperature and energy consumption, minimal use of organic solvents, easier wastewater treatment, significantly reduces environmental pollution	Requires additional degumming compared to physical and chemical oil extraction methods
Low-temperature non-organic solvent extraction	Protects heat-sensitive components in oils, suitable for extracting heat-sensitive oil components	Extraction efficiency may be low; process optimization is required to improve efficiency
Supercritical CO_2_ extraction	Can extract at room temperature, preventing the oxidation and volatilization of heat-sensitive substances; CO_2_ is inexpensive, easy to prepare, and recyclable	High equipment cost, making it less suitable for large-scale production

In addition to organic solvent extraction, various alternative methods for oil extraction have been investigated, including enzyme-assisted water extraction, low-temperature non-organic solvent extraction, high hydrostatic pressure (HPP) extraction, and scCO_2_ extraction (Ahmet et al., 2020; Choi et al., 2017). Enzyme-assisted water extraction employs mechanical disruption in conjunction with enzymatic hydrolysis to rupture cell membranes, thereby facilitating the aqueous extraction of lipids. Low-temperature non-organic solvent extraction exploits non-organic solvents (e.g., water or alcohol) under strictly controlled low-temperature conditions, allowing for the solubilization of lipids through a combined effect of physical and chemical mechanisms. scCO_2_ extraction capitalizes on the tunable solubility and diffusivity of scCO_2_ at elevated pressures (8–30 MPa) and temperatures (35–60℃) to enable lipid extraction, which is followed by separation achieved through depressurization or modulation of temperature.

### Protein extraction

Current methodologies for protein extraction from insects encompass various techniques, primarily including alkali-based extraction, salt-based extraction, protease-based extraction, and Tris-HCl buffer-based extraction (Mahfuzur et al., 2023). The diversity in these methodologies is attributed to the significant anatomical variability and the distinct physicochemical properties of proteins across different insect species, thereby necessitating customized strategies to enhance extraction efficiency (Miranda et al., 2024; Shi et al., 2024) (Table 3).

**Table 3:** Comparison of different insect protein extraction methods

Method	Advantages	Disadvantages	Yield	Economic Feasibility	Application Scope
Alkali extraction method	Simple operation Cost-Effectiveness Minimal equipment requirements	Protein denaturation Long processing time Poor selectivity	Medium (60–80%)	Low extraction cost High cost of wastewater treatment	Large-scale industrial production
Salt extraction method	Does not alter protein internal structure	Salt residue	Medium (50–75%)	Low energy consumption Dependence on salt prices	Extractions with specific protein structural requirements
Protease dissolution method	Short extraction time No harmful by-products High purity Environmentally friendly	Sensitive to reaction conditions	High (70–85%)	Hight cost of enzymes Low energy consumption	Large-scale experimental extractions
Tris-HCl extraction method	High solubility of proteins Minimal damage High protein purity Wide compatibility	Solvent residue	Medium (55–70%)	High extraction cost	Small-scale, high-precision experimental extractions
Supercritical CO_2_ extraction	No solvent residue High purity	Small processing capacity	Low (30–40%)	High equipment cost High energy consumption	Small scale high value-added production
Enzyme-assisted extraction	High selectivity Environmentally friendly	Poor stability	High (75–90%)	Hight cost of enzymes Low energy consumption	Medium to large-scale experimental extractions
Ultrasonic or microwave-assisted extraction	Fast and efficient Environmentally friendly	Protein aggregation Enlarge difficulties Protein denaturation	Medium (65–80%)	High equipment maintenance costs	Laboratory to pilot scale Often used with other extraction methods
Dry fractionation technology	Environmentally friendly	Low purity	Medium (60–75%)	High equipment investment	Large scale continuous production

### Alkali-based extraction

The alkali extraction method functions through two principal mechanisms: the disruption of intermolecular hydrogen bonds within protein structures and the dissociation of polar functional groups under elevated pH conditions (pH 9–11). These processes facilitate the detachment of proteins from associated biomolecules (Wittmann et al., 2024). Subsequent to homogenization and centrifugation, the pH of the resulting supernatant is adjusted to the protein’s isoelectric point, thus inducing precipitation. This technique is extensively employed for the isolation of proteins from *Tenebrio molitor*, *Bombyx mori*, *Mythimna separata*, and *Hermetia illucens*. For instance, Caligiani et al. (2018) demonstrated the efficacy of protein extraction from *Hermetia illucens* prepupae using optimally established parameters: 1 mol/L NaOH solution was utilized for 1 h at a temperature of 40℃, followed by precipitation using 10% trichloroacetic acid (TCA) in acetone at a ratio of 1:1 (v/v). The resultant extraction yield was quantified at 73 ± 2.5% (w/w dry weight basis).

### Salt-based extraction

The salt-based extraction method is predicated on the modulation of ionic strength to influence protein solubility. At low salt concentrations (ranging from 0.1 to 1.0 mol/L), the salting-in effect serves to enhance protein solubility by mitigating the electrostatic repulsions that exist between peptide chains. Conversely, at elevated salt concentrations exceeding 1.5 mol/L, the salting-out phenomenon is activated, resulting in the dehydration of hydration shells, which subsequently leads to protein aggregation and precipitation (Hall et al., 2016). This methodology effectively exploits these divergent solubility behaviors to facilitate the sequential extraction and fractionation of target proteins. NaCl solutions are the most commonly utilized, with their concentrations optimized according to the charge density and hydrophobicity of the proteins in question.

For instance, the salt-based extraction method has been successfully employed for the isolation of proteins from *Musca domestica* larvae and *Periplaneta americana* (American cockroach). Wang et al. (2017) utilized an orthogonal array design to ascertain optimal extraction parameters for *Periplaneta americana*, identifying the conditions as 1 mol/L NaCl at 40℃ over a duration of 2 h, resulting in a protein recovery of 7.67% (w/w). Notably, this recovery yield is significantly lower than that achieved through enzymatic (58.87%) and alkaline (37.96%) extraction methods.

### Protease-based extraction

The Protease-based extraction method employs one or more biological enzymes to enzymatically digest and dissolve cell membranes, thereby facilitating the extraction of intracellular proteins from insect cells. A crucial point of this methodology is the selection of enzymes that exhibit specificity for insect-specific structural proteins, such as chitinases, which target the degradation of the exoskeleton. This selection is informed by the distinct anatomical and biochemical characteristics inherent to different species (Mintah et al., 2019).

Proteases have demonstrated effectiveness in degrading proteinaceous matrices at low concentrations (ranging from 0.1 to 1.0 Ug⁻¹). However, excessive enzyme loading, exceeding 2.0 Ug⁻¹, may induce over-hydrolysis, resulting in peptide fragmentation and a concomitant loss of functional properties (Shuangmei et al., 2023). This technique is commonly employed for the isolation of proteins from various insect sources, including *Tenebrio molitor*, *Bombyx mori*, and *Musca domestica*. For instance, Li et al. (2011) optimized the hydrolysis parameters for *Tenebrio molitor* utilizing an orthogonal array design, establishing optimal conditions at 50℃, pH 1.5, a hydrolysis duration of 6 h at 8000 Ug⁻¹ enzyme dosage. Under these refined conditions, the protein extraction yield achieved was 51.24% on a dry weight basis.

### Tris-HCl buffer-based extraction

The Tris-HCl buffer extraction method leverages the pH buffering capabilities of Tris (hydroxymethyl) aminomethane to maintain protein solubility within the physiological pH range of 7.0–9.0. By stabilizing the solution pH at 7.2–7.4, this technique effectively preserves the native conformation of soluble proteins, thereby minimizing denaturation. This characteristic renders it particularly advantageous for high-precision experiments that necessitate the retention of protein bioactivity and functionality (Carrio-Sá et al., 2024). The Tris-HCl buffer system plays a critical role in mitigating pH fluctuations during homogenization and centrifugation, ensuring that the solubility of impurities is minimally affected. This extraction method is widely utilized for the isolation of proteins from *Tenebrio molitor* and *Hermetia illucens* larvae. Nevertheless, the practical application of this technique for large-scale production is hindered by relatively low extraction yields, which typically range from 8.5% to 15.3% (w/w) as reported by Xu et al. (2014).

### Other extraction method

Recent advancements in insect protein extraction have moved beyond conventional methods, with research focusing on innovative techniques including scCO_2_ extraction, enzyme-assisted extraction, ultrasonic or microwave-assisted extraction and dry fractionation technology. For instance, scCO_2_ extraction harnesses the solvating power of supercritical fluids to selectively isolate heat-sensitive proteins (e.g., silkworm pupa protein) at mild temperatures, minimizing structural denaturation. Enzyme-assisted extraction utilizes the catalytic specificity of enzymes to break through insect structural barriers, achieving efficient protein release and functional retention under mild conditions. Ultrasonic or microwave-assisted extraction relies on cavitation-induced shear forces or dielectric heating effect to destroy the organizational structure, thereby enhancing protein release efficiency through intensified mass transfer (Khushar et al., 2023). Dry fractionation technology enables the prioritized destruction of chitin-protein complexes and the preservation of protein backbone structures by controlling the pyrolysis temperature gradient under anaerobic conditions, while separating interfering components such as fat (Sweers et al., 2022).

For example, Fan et al. (2024) introduced a novel protein extraction protocol for larvae of *Musca domestica* by combining UAE with enzymatic hydrolysis. This integrative methodology achieved a bioactive peptide recovery rate of 82.56 ± 0.42%, demonstrating an 8.25% improvement in peptide yield and a 14.83% enhancement in DPPH radical scavenging capacity compared to conventional extraction methods. The superior performance observed in this study is attributed to ultrasonic cavitation, which induces structural remodeling and protease cleavage sites exposing of *Musca domestica* protein.

### Peptide separation and purification

The extraction process for insect peptides shares similarities with protein isolation but requires additional optimization due to their smaller molecular weight (0.1–100 kDa) and typically low abundance. Peptides expression is often induced prior to extraction, particularly for AMPs and other immune-related peptides.

Common methods for insect peptides extraction include enzymatic hydrolysis, membrane separation technology, gel filtration chromatography, reverse-phase high-performance liquid chromatography (RP-HPLC), among others (Table 4). For instance, Liu et al. (2024a, b, c) induced AMPs from the *Periplaneta americana* using *Escherichia coli* (*E. coli*) challenge and subsequently purified them via ammonium sulfate precipitation. The highest protein concentration (1.673 ± 0.036 mg/mL) was observed 48 h post-induction. Similarly, Hu et al. (2024) optimized ultrasonic-assisted enzymatic hydrolysis parameters for *Hermetia illucens* AMPs extraction, achieving a 26.16 ± 0.94% yield under conditions of 53 W ultrasonic power, 23 min sonication time, 52℃ hydrolysis temperature, and pH 7.3.

**Table 4:** Comparison of different insect proteins and peptides purification methods

Method	Advantages	Disadvantages	Yield	Economic Feasibility	Application Scope
Enzymatic hydrolysis	Mild conditions Preserve bioactivity High specificity	Requires enzyme ratio optimization Over-hydrolysis may fragment peptides	75–90%	High enzyme cost	Suitable for high-value products like antimicrobial peptides
Microbial fermentation	Improves flavor and shelf life	Long cycle (24–96 h) Prone to contamination Yield depends on strains	60–75%	Low cost	Suitable for large-scale feed/primary food production
In vitro gastrointestinal digestion	Simulates in vivo digestion	Affected by digestive enzyme activity	50–70%	Suitable for research-oriented functional peptide screening	Nutraceuticals Preclinical drug development
Membrane separation technology	Pure physical process Mild conditions Simple operation	Inability to separate small molecules	50–80%	Low equipment cost	Suitable for large-scale purification Often used with other purification methods
Gel Filtration chromatography	Mild separation preserves activity	Slow flow rate Small column capacity Relying on molecular size differences	60–80%	Requires chromatography equipment	Suitable for laboratory-level Laboratory isolation of bioactive peptides
RP-HPLC	High purity	High solvent consumption High equipment investment	50–70%	Only suitable for pharmaceutical/high-value	Pharmaceuticals Cosmetics
Chemical synthesis	Precise sequence control Impurity-free	Error-prone for long peptides	30–60%	Extremely high cost	Suitable for short peptides (< 20 aa) Only suitable for drug development
Recombinant DNA technology	High expression yield	Host cells may lack post-translational modifications Complex purification steps	70–90%	Requires genetic engineering equipment	Suitable for industrial mass production Suitable for complex peptides

### Quality control of insect proteins and peptides

The quality assessment of insect proteins and peptides necessitates comprehensive evaluation across physicochemical indicators, structural characteristics, and safety parameters. Physicochemical analyses include protein content determination via the Kjeldahl method or BCA assay, requiring crude protein to be ≥ 70% on a dry basis, and purity assessment through SDS-PAGE (verifying main band proportion ≥ 95%) and HPLC (detecting host protein residues ≤ 0.1%). Structural characterization requires confirmation by mass spectrometry (MS) with molecular weight deviation limited to ≤ ± 0.1 Da (Some studies also use specific or in-house databases for edible insects for more precise identification) (Fan et al., 2021), complemented by functional activity analyses (e.g., antioxidant activity with IC_50_ ≤ 50 µg/mL). For example, Giulia et al. (2020) identified three insect specific peptide biomarkers, SVSPEAAAELR, VWPLLSNVALSAPLVR, and VWPLLPNVAVAALPVR, using LC-MS/MS. This detection method addresses the limitations of traditional quality control measures in processed products through screening of highly specific labeled peptides, verification by dual mass spectrometry, and adaptability to complex matrices. Safety testing encompasses microbial indicators (total bacterial count ≤ 1,000 CFU/g; Salmonella non-detectable) and heavy metal residues (lead ≤ 0.02 mg/kg; arsenic ≤ 0.01 mg/kg) (Dewi et al., 2025). In addition, regulatory frameworks mandate the European Food Safety Authority (EFSA) allergenicity assessment and “novel food” labeling for insect proteins in the EU; the U.S. FDA requires GRAS certification for insect proteins used in pet food; and ISO 22,000 and Halal certification govern end-to-end production chain compliance.

## Functional components of insect proteins and peptides

Insects serve as a rich source of high-quality proteins, bioactive peptides and essential amino acid, which possess multifaceted biological activities, including blood pressure modulation, antioxidative effects, and immune system enhancement (Table 5). A pertinent example is the AMPs derived from *Bombyx mori*, notably Cecropin B, which exhibited significant antitumor properties (Tornesello et al., 2020). These peptides not only play effective roles in murine models (Ramanathan et al., 2018), but also possess the potential to inhibit the proliferation of specific tumors and hepatitis viruses in clinical settings. Furthermore, insect peptides demonstrate immunomodulatory effects (Gessica et al., 2023). The cyclic GMP-AMP (cGAMP) derived from *Bombyx mori* activates antiviral immunity by disrupting the interaction between BmAsp8L and BmSTING, leading to the generation of effective antiviral signals and offering protection to insect cells against viral infections (Hua et al., 2018).

**Table 5:** Common functional substances and their effects on insect protein peptides

	Effect	Source	References
AMPs	Antibacterial, antiviral, antitumor	*Bombyx mori*,* Hermetia illucens*	Hu and Sun (2023) Li & Cui (2011)
C-type lectins	Defense against microbial invasion	*Bombyx mori*,* Musca domestica*	Huseynli et al. (2023) Li et al. (2017)
Antioxidant enzymes	Improve metabolism, antibacterial	*Tenebrio molitor*,* Delia antiqua*	Liu et al. (2019)
Fibrinolytic active proteins	Protect hypoxic cells, anti-thrombosis	*Mesobuthus martensii*,* Myrmeleon formicarius*	Ban et al. (2018) Xu et al. (2019)

Additionally, fibrinolytic peptides extracted from scorpion venom have been shown to protect endothelial cells from damage caused by hypoxia (Brazón et al., 2014), while analgesic peptides from the same venom exhibited notable pain-relieving and anti-aging activities (Ban et al., 2018). In terms of practical applications, AMPs, insect lectins, and other peptides derived from *Bombyx mori*, *Musca domestica*,* Tineola bisselliella*, and other species have been utilized in the development of antiviral and antibacterial therapeutics (Xuan et al., 2023; Zhou et al., 2024).

Researchers have compared the biological activity of insect peptides with that of peptides from traditional sources. For instance, it has been reported that the ACE inhibitory activity of silkworm pupae (IC_50_ = 9.84 µg/mL) was significantly higher than that of soybean peptides (IC_50_ ≈ 10–50 µg/mL) and whey protein hydrolysates (IC_50_ ≈ 10–30 µg/mL). The DPPH radical scavenging activity of yellow mealworm hydrolysates (EC_50_ = 0.32 mg/mL) exceeded that of soybean peptides (EC_50_ ≈ 1–3 mg/mL) and approached that of fish peptides (EC_50_ ≈ 0.1–0.5 mg/mL) (Ma et al., 2023). This is because insect peptides (such as silkworm pupa peptide Lys-His-VAL and cricket peptide YAN) are rich in hydrophobic amino acids, which can form strong coordination effects (such as hydrogen bonds and van der Waals forces). These results indicate that the bioactivity of insect-derived peptides has approached or even surpassed that of most traditional active peptide sources.

### AMPs

AMPs are short cationic peptides ranging from 10 to 50 amino acids, recognized for their broad-spectrum antimicrobial properties and diverse structural motifs. These small molecules are secreted by various organisms as a defensive response to environmental stressors, functioning as integral components of innate immune system (Yi et al., 2014). A primary antibacterial mechanism of action for antimicrobial AMPs involves disrupting the plasma membrane of target pathogens. Most AMPs exhibit amphipathic properties, comprising hydrophobic and hydrophilic domains, which enable their insertion into the lipid bilayer of microbial pathogens. This interaction facilitates processes such as pore formation or compromise of membrane integrity, ultimately leading to cellular dysfunction or lysis (Nahas et al., 2022). Specifically, this mechanism disrupts the pathogen’s ability to maintain osmotic equilibrium, culminating in cellular lysis. For instance, AMP-17—a novel AMP derived from *Musca domestica*—induces 21.7% degradation of the Candida albicans cell wall, demonstrating its efficacy in compromising microbial structural integrity (Ma et al., 2020). Due to their significant biological activities, non-toxicity, and low risk of resistance development, AMPs have emerged as promising candidates for antibacterial agents and feed additives.

For instance, research conducted by Hu & Sun, (2023) extracted and purified AMPs from *Hermetia illucens* larvae, demonstrating a notable inhibition zone of 12.68 ± 0.72 mm on Mueller-Hinton agar against *Staphylococcus aureus* (*S. aureus*). Similarly, Li et al. (2020) observed a selective antimicrobial spectrum, wherein crude extracts effectively inhibited *S. aureus* but exhibited minimal activity against *E. coli*. Zhang et al. (2023) documented a minimum inhibitory concentration (MIC) of 2.5 mg/mL for *Hermetia illucens*-derived AMPs against *Salmonella enterica*, with additional inhibition of *Aspergillus niger* spore germination at this concentration.

### CTLs

Lectins function as pattern recognition receptors (PRRs) within the insect innate immune system, playing pivotal roles in microbial surveillance and the activation of immune responses. A prominent subgroup of insect lectins is the CTLs, which are carbohydrate-binding proteins whose activity is contingent upon the presence of calcium ions. In model organisms such as *Drosophila melanogaster* and *Bombyx mori*, CTLs localized on the membranes of hemocytes serve as crucial sensors of immune stimuli. These lectins are implicated in the modulation of cytoskeletal organization, which induces morphological changes in the cells, such as rounding or elongation, as demonstrated by Xiu et al. (2015). This suggests a potential involvement of CTLs in various cellular activities and the regulation of metabolic processes. In the human body, CTLs may activate the phagocytic function of neutrophils, macrophages, and other phagocytic cells to exert antibacterial effects by recognizing lipopolysaccharides of Gram-negative bacteria or peptidoglycans of Gram-positive bacteria. In addition, CTLs can bind to abnormal glycosylation structures on the surface of tumor cells (such as sialyl Tn antigens, Lewis X), enhance the killing effect of natural killer cells and cytotoxic T cells, thereby exhibiting the potential to target tumor cells. However, due to the possibility that the heterologous sugar chains of CTLs may be recognized by the human immune system as “non-self” components, there is a risk of inducing allergic reactions or autoimmune responses, and the clinical translation of their biological activity still requires further investigation.

Further investigations by Zhou et al. (2018) characterized two CTLs (MdCTL1 and MdCTL2) derived from *Musca domestica*, revealing their antiviral properties against the human pathogenic RNA virus H1N1, thereby indicating their prospective application as therapeutic agents for influenza. Additionally, Takase et al. (2009) identified three novel CTLs from *Bombyx mori*, with structural analyses highlighting their Ca^2+^-dependent affinity for lipopolysaccharide (LPS), thereby confirming their instrumental role in the recognition of Gram-negative bacterial pathogens.

### PPO

PPO, commonly referred to as tyrosinase, is a copper-containing oxidase that exists as a tetrameric complex across a range of organisms, including insects, plants, and fungi (Liu et al., 2024a, b, c). In invertebrates, particularly insects, PPO plays a crucial role as an immune protein. Additionally, PPO is involved in other essential processes such as exoskeleton hardening, eggshell formation, pigment deposition, and molting. For instance, the expression level of PPO_4_ in *Anopheles mosquitoes* is significantly upregulated following *Plasmodium* infection, contributing to the blackening envelope reaction (Zhu et al., 2025). In vitro, PPO exists as a zymogen (pro-PPO). It is activated by proteases (e.g., chymotrypsin) or 30% ethanol, exposing Cu^2+^ in the active site. Cu^2+^ catalyzes the hydroxylation of monophenols (e.g., tyrosine) to form o-diphenols, which are further oxidized to o-quinones with the generation of H_2_O_2_. O-quinones polymerize into melanin, which physically entraps bacteria, while H_2_O_2_ disrupts pathogen cell membranes through oxidative damage, collectively achieving bacteriostatic effects. But its human application needs to break through the bottleneck of immunogenicity and stability (Wu et al., 2024).

Shao et al. (2012) conducted a characterization study of PPO activity in the hindgut of *Bombyx mori*, highlighting its significant function in the initiation of melanization processes within hindgut fecal material. This melanization not only acts to inhibit bacterial proliferation but also plays a role in modulating the microbial composition of the gut. Their findings further substantiate the broad-spectrum antibacterial properties of PPO, demonstrating its efficacy against a range of pathogenic bacteria, including *E. coli*, *Salmonella typhimurium*, *S. aureus*, and *Bacillus subtilis*. These insights underscore the multifaceted role of PPO in both immune defense and gut health.

### Fibrinolytic active proteins

Fibrinolytic proteins, including plasmin and plasminogen activators, are serine proteases that specifically degrade fibrin clots into soluble fibrin degradation products (FDPs). These enzymes exhibit potent fibrinolytic activity while maintaining minimal hemolytic effects, positioning them as promising thrombolytic candidates. By hydrolyzing fibrin cross-links, fibrinolytic enzymes play a critical role in hemostasis, preventing pathological thrombus formation and maintaining vascular patency (Zhong et al., 2024).

Zhang et al. (2024) isolated fibrinolytic proteins from *Megasoma elephas* larvae using ammonium sulfate precipitation. They demonstrated that these proteins inhibited cancer cell growth. Xu et al. (2019) optimized fibrinolytic protein extraction from *Myrmeleon formicarius* using ammonium sulfate fractionation (37℃, pH 6.0). Activity assays showed maximal fibrinolytic activity (1,200 ± 150 U/mg) in the 35%–70% saturation fraction, surpassing urokinase (100 IU/mL) by 2.3-fold. Zhang et al. (2024) validated the strong immunogenicity and certain antithrombotic effects of fibrinolytic proteins from *Tenebrio molitor*.

### Challenges in biological activity research

Although the bioactive components derived from insects have antioxidant, antibacterial, antithrombotic, and other biological activities, research on their biological activities still has some limitations and variability. Different insect species, peptide sequences, or extraction methods will lead to different biological activities. For example, under the same conditions, silkworm pupae and crickets were hydrolyzed using a two-step method with gastric protease and trypsin to obtain ACE inhibitory peptides. The bioactivity of silkworm pupae-derived peptides (IC_50_ = 8.3 µg/mL) was significantly higher than that of cricket-derived peptides (IC_50_ = 20.74 µg/mL). This is because silkworm pupae protein is rich in hydrophobic amino acids (such as Pro, Leu), which form peptide segments (such as Ala-Ser-Leu) that bind more tightly to the ACE active pocket (Ma et al., 2023). In addition, compared to traditional enzymatic hydrolysis (IC_50_ = 0.20 mg/mL), the ACE inhibitory activity of cricket protein hydrolysates was significantly increased after microwave-assisted enzymatic hydrolysis (IC_50_ = 0.096 mg/mL). This is because microwave-assisted enzymatic hydrolysis disrupts the protein’s tertiary structure, exposes more hydrophobic amino acids (such as Tyr and Val), and enhances the binding between peptides and the ACE active center (Zn^2+^ site) (Ma et al., 2023). The divergence in antibacterial specificity between apidaecin and abaecin in bees stems from differences in proline distribution and peptide length. Apidaecin (18-mer) contains six prolines concentrated at the N-terminus (Pro4-Pro5-Pro6), with a C-terminal Lys-Arg motif. Its N-terminal proline cluster forms a rigid structure that targets specific channels in the outer membrane of Gram-negative bacteria, mediating energy-dependent transmembrane transport to disrupt bacterial protein synthesis and specifically inhibit pathogens like *E. coli*. In contrast, Abaecin (34-mer) harbors ten more diffusely distributed prolines and a C-terminal Gly-Gly motif. The flexible proline arrangement enables broader interaction with bacterial membranes, expanding its antibacterial spectrum to Gram-positive species such as *Enterococcus faecalis*, with an inhibitory concentration (MIC) tenfold lower than that of Apidaecin. This disparity highlights how proline density, spatial distribution, and peptide length dictate antibacterial specificity and potency by influencing conformational rigidity and membrane interaction patterns (Yi et al., 2014).

Therefore, to improve scientific transparency and research reliability, the importance of standardizing insect active ingredients must be emphasized. In the short term, it is necessary to promote the standardization of sample preparation and extraction methods and conduct cross-species activity validation (e.g., selecting peptides with similar functional domains such as the antimicrobial peptides cecropin and defensin, and comparing their antimicrobial/antitumor activities in the same model to clarify structure-function relationships (Moehle et al., 2021). In the long run, establishing a database of insect peptide sequences and active ingredient data, promoting the adoption of international standards, and achieving mutual recognition of global biological activity research data are essential (Zhang et al., 2011).

## Current applications of insect proteins and peptides

**Fig. 3:**
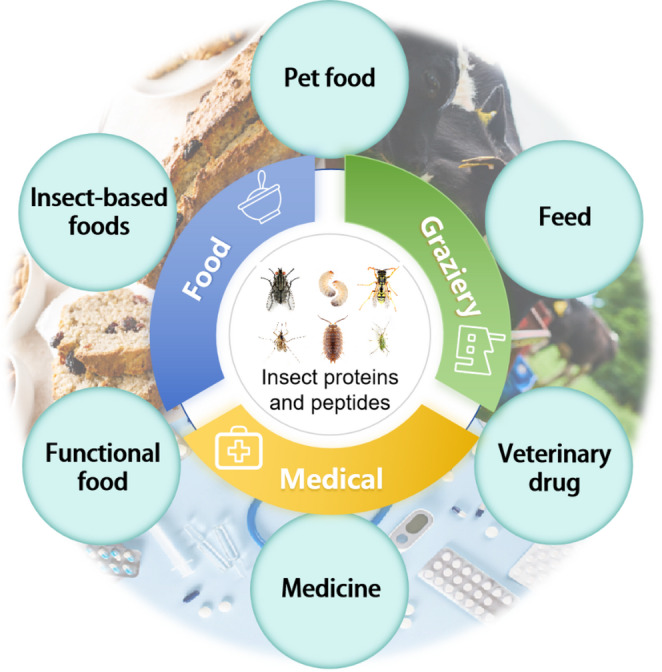
Application of insect proteins and peptides

Insect proteins and peptides are gaining traction in food, feed, and pharmaceutical industry. In the food industry, insect protein extracts enhance nutritional value, flavor, and texture. In feed industry, their high nutritional content makes them a sustainable alternative to fishmeal and soybean protein (Hong et al., 2020). In pharmaceutical industry, insect peptides and proteins can be applied for new vaccines, antibiotics, and therapeutic solutions. As their applications grow, the recognition of insects as a valuable protein resource will increase (Fig. 3).

Advantages of Applications: Sustainable Production & Nutritional Benefits.

The burgeoning demand for protein on a global scale is being significantly influenced by advancements in the food, feed, and pharmaceutical industry. Insects are emerging as a viable sustainable source of protein, garnering attention due to their environmental sustainability and superior nutritional profiles in comparison to traditional livestock. Insect rearing systems exhibit remarkable land-use efficiency—requiring merely 15 m^2^/kg of protein for *Gryllodes sigillatus* compared to 50 m^2^/kg for pigs and 200 m^2^/kg for cattle—alongside lower production costs and a reduced carbon footprint (Goodland, 2013). The biological characteristics of insects—characterized by high fecundity (producing between 500 and 1,000 offspring per female), rapid larval growth (2–4 weeks), and short generational cycles (6–8 weeks)—further substantiate their potential for enhanced production efficiency (Wendin & Nyberg, 2021). Additionally, the capacity of most insects to utilize organic waste as a substrate for growth not only facilitates waste decomposition but also promotes the principles of a circular economy (Ojha et al., 2020).

Recent studies, such as that conducted by Kröncke & Benning Rainer, (2023), have evaluated the performance of *Tenebrio molitor* larvae reared on diets with varying protein concentrations. The findings indicated that larvae receiving 80% pea protein isolate achieved a protein content of 74.1 ± 0.2% (dry matter), representing a 3.72-fold increase over pork and a 5.70-fold increase over chicken eggs. Moreover, the feed conversion ratio (FCR) for these larvae was recorded at 3.7 ± 0.9, while the fat content remained significantly lower at 20.3 ± 0.3% (w/w), or 50.4% of that found in pork. Concurrently, Zielińska & Pankiewicz, (2023) emphasized the potential of *Gryllodes sigillatus* (crickets) as sustainable protein sources for functional food applications. Their analysis revealed that cricket powder contained 73.68% protein DM and demonstrated substantial antioxidant activity against DPPH radicals, yielding a trolox equivalent (TE) value of 2.179 mmol TE/100 g. These findings illuminate the dual functionality of edible insects as both high-quality protein sources and bioactive food components, which can enhance nutritional density and confer health-promoting benefits.

### Applications in the food industry

In various regions of Africa, Asia, and South America, insects have been part of traditional diets and are often prepared through methods like drying and fermentation (Mishyna et al., 2021). Despite their nutritional benefits, entomophagy faces cultural resistance in Western societies, where insects are often seen as unappealing or unsanitary (Wendin & Nyberg, 2021). However, growing concerns about sustainability and food security have led to increased interest in insect-based proteins as eco-friendly alternatives to traditional livestock.

Scholliers et al. (2020) partially replaced pork (5–50%) in the production of cooked sausages using *Zophobas morio* beetle larvae powder. Compared to sausages made from pork, using 5%−10% insect powder helps reduce cooking losses and provides sufficient emulsifying stability. However, excessive increase in the amount of insect powder used to replace meat can lead to a decrease in the texture characteristics of the obtained product. In cereal applications, Andrea et al. (2019) demonstrated that fortifying white bread with 10% *Tenebrio molitor* powder increased protein content from 8.91 ± 0.23% to 11.55 ± 0.33%. This formulation also resulted in statistically significant increases in essential amino acid profiles, with tyrosine (68%), methionine (60%), isoleucine (53%), and leucine (46%) showing the highest enhancements compared to unfortified bread.

### Applications in the feed industry

Insect protein is increasingly used in pet feed. Ye et al. (2023) successfully incorporated insect AMPs into immune-enhancing feed additives for aquaculture, demonstrating that these additives significantly improved the immunity of sea cucumbers. In early 2019, the UK pet food company Yora launched a new type of dog food made from *Hermetia illucens* protein. This dog food not only offered a novel protein source but was also more easily digestible compared to traditional meat-based options, making it ideal for pets with sensitivities to conventional proteins. By the end of that year, the Russian pet food company Limkorm established the country’s first processing facility for *Hermetia illucens* larvae pet food, boasting an annual production capacity of 30,000 tons (Zych, 2024).

### Applications in the pharmaceutical industry

Escalating antibiotic resistance driven by overuse has emerged as a critical threat to global public health, with estimates projecting 1.3 million annual deaths by 2023 (Alkorta & Garbisu, 2024). This urgency necessitates the development of novel antimicrobial agents. AMPs derived from insects represent a promising alternative, characterized by their small molecular weight, thermal stability, broad-spectrum activity, and reduced resistance potential compared to conventional antibiotics (Ferrazzano et al., 2012). Beyond AMPs, insect-derived bioactive proteins and peptides—including CTLs, PPOs, fibrinolytic proteins, and antifreeze proteins(AFPs)—exhibit multifunctional properties with significant medical applications.

Matos et al. (2021) elucidated the substantial antioxidant properties of protein extracted from *Gryllodes sigillatus*, highlighting the potential of enzymatic hydrolysis to augment these antioxidant activities. Concurrently, Lee et al. (2023) characterized a novel protein, designated Amwaprin, found in the venom of *Apis mellifera*, which exhibited both microbicidal and anti-elastolytic effects. Furthermore, Roberta et al. (2025) conducted an investigation into various peptide fractions derived from *Hermetia illucens*, assessing their impact on AGS and KATO III gastric cancer cell lines. Their findings revealed that specific peptide fractions, particularly those derived from infections with *E. coli* and *Micrococcus flavus*, effectively inhibited tumor cell proliferation through the induction of apoptotic pathways and cell cycle arrest. Collectively, these studies underscore the necessity for further investigation into insect-derived antimicrobial peptides as promising candidates for the development of novel anticancer therapeutics.

In cryomedicine, insect-derived AFPs exhibit ice crystal growth inhibition and freezing point depression through thermal hysteresis activity. Jevtić et al. (2022) and Varga et al. (2022) independently isolated ApAFP752 from *Anatolica polita* for cryopreservation of *Xenopus laevis* embryos and mammalian cells, respectively, results indicate that both intra and extracellular ApAFP752 significantly improved cell survival after freeze/thaw. While these proteins demonstrate cytoprotective effects on human gametes and embryos in laboratory settings, their capacity to enable long-term organ preservation remains investigational. Current evidence suggests AFPs may mitigate cryodamage to hepatic tissues, but translation to clinical organ storage requires further validation (Matsumura & Hyon, 2009; Ryeol et al., 2015).

## Main issues and challenges in the development of insect proteins and peptides

### Low consumer acceptance

Low consumer awareness and acceptance of insect proteins and peptides products remain a critical challenge. Most consumers resist eating insects or their derived products. According to research by Wade & Hoelle, (2020), “neophobia” is the main reason why Australians refuse to eat insects, in addition. Factors such as environmental issues, social and cultural background, and the processing methods of products can also affect consumers’ acceptance level to a certain extent (Mina et al., 2023; Roma et al., 2020). How to improve public acceptance of insect proteins and peptides products will be a key issue in the development of insect protein resources. Taking crickets as an example, the protein derived from crickets contains high levels of umami amino acids such as glutamic acid, endowing it with a flavor profile reminiscent of seafood like shrimp and crab. Meanwhile, it exhibits a natural odor akin to shrimp shells and crustaceans, intermingled with subtle grassy or earthy undertones. The chitin and chitosan in cricket exoskeletons may impart a slight bitterness, while melanin in the exoskeletons gives cricket powder a dark brown hue. These inherent characteristics are transferred to food products upon the addition of cricket powder. Cappelli et al. (2020) conducted a sensory evaluation of Frankfurt sausage incorporating cricket powder as a meat substitute, assessing five key dimensions: appearance, color, texture, taste, and aroma. The study revealed that low-level incorporation of cricket powder did not impair the organoleptic properties of the food, however, higher inclusion levels were associated with a deterioration in multiple sensory attributes. When excessive cricket powder is used, additional processing of the cricket powder becomes necessary to mitigate these adverse effects. In addition, a survey conducted on 363 Norwegian consumers (Ribeiro et al., 2022) showed that consumers had an acceptance rate of 3.2 for direct consumption of insects (evaluated on a 7 - point anchoring scale, ranging from 1 point - completely rejected to 7 - completely accepted), 3.9 for protein bars containing insects, and 5.0 or higher for animal products (Poultry, Pork, Beef, Fish) produced from livestock fed with insect feed.

Consumer preference trials demonstrate that formulated insect-based products exhibit higher purchase intent compared to whole-insect formulations, particularly when insect content is maintained below 15% by weight (Wendin & Nyberg, 2021). Controlled sensory evaluation trials further indicate that gradual exposure to insect-enriched foods through familiar product formats reduces neophobia in a dose-dependent manner, suggesting that incremental integration represents a viable strategy for mainstream market penetration. Incorporating insect biomass into processed meat products (e.g., hamburgers, sausages) and cereal-based goods (e.g., pasta, bread) can effectively mask entomological sensory attributes (Cavalheiro et al., 2023). This approach capitalizes on the sensory familiarity of conventional food matrices while leveraging the nutritional benefits of entomological ingredients, thereby addressing both hedonic and health-related consumer concerns (Mbi et al., 2023). In addition, in terms of cultural promotion, it is equally important to emphasize the nutritional and environmental benefits of insect food (Zielińska & Pankiewicz, 2023), and eliminate consumer cultural biases through activities such as insect food festivals and cooking courses (such as the insect food market in Bangkok, Thailand) (Van & Rumpold, 2023).

### The challenge of economic feasibility

While the cultivation costs of insects remain significantly lower than those of traditional livestock—for instance, the commercial production cost of mealworm protein is USD 7.53 per kg, compared to USD 59.04 per kg for edible pork protein—the economic viability of insect protein production is still constrained by processing complexity and technological immaturity (Liceaga, 2025). Consequently, this has resulted in current insect protein products being priced 80% to 300% higher than traditional protein products (Omuse et al., 2024). Laboratory-scale techniques such as non-denaturing drying and extraction, although suitable for small-scale research, are ill-suited for industrial applications due to high energy demands and scalability limitations. For instance, in the current aquaculture market, black soldier fly larvae meal incurs production costs of $2,000–$3,000 per metric ton, remaining uncompetitive with fish meal ($1,870/ton) (Selvaraj et al., 2024). This cost disparity stems primarily from fragmentation in the insect protein processing chain—including inefficient integration of larval separation, sterilization, and dehydration—and the absence of standardized quality grading systems, which perpetuates market pricing ambiguity (Aysan et al., 2024). Production costs for cricket protein have declined approximately 30% annually since 2018 as operations scale and efficiencies improve. Industry analysts project price parity with conventional animal proteins for certain applications by 2026, potentially triggering more rapid adoption in mainstream food products.

In addition, the improvement of the supply chain will further promote the economic feasibility and scalability of insect protein production. For example, the EU’s Horizon 2020 project “SUSINCHAIN” (Sustainable Insect Chain) brings together multiple stakeholders such as farmers, processors, retailers, research institutions, and governments to build a complete supply chain loop through knowledge-sharing mechanisms, technology standardization systems, and raw material recycling models. This project breaks through the market barriers and technological bottlenecks faced by the traditional insect industry, improves resource recycling efficiency while reducing comprehensive production costs, and enhances the overall risk resistance of the supply chain by establishing a regional emergency supply chain system (Veldkamp et al., 2022). Long-term insect proteins and peptides market penetration in human food applications will hinge on supply chain optimization, advancements in processing technology, and consumer acceptance, underscoring the need for interdisciplinary collaboration across research, industry, and policy domains (Song et al., 2023).

### Safety risk uncertainties

Uncertainties persist regarding the safety risks associated with consuming insect proteins and peptides, which encompass three primary categories: biological, chemical, and allergic risks (Gravel, 2020). For biological risks, viruses that are pathogenic to insects do not pose a significant risk to humans, as genetic differences between humans and insects make them unique to invertebrates; moreover, basic processing methods (e.g., freezing, cooking) can further mitigate these risks by inactivating potential biological contaminants.

According to the European Food Safety Authority (EFSA), the most critical chemical pollutants of concern include heavy metals (e.g., cadmium, mercury, lead, arsenic), as well as accumulated environmental contaminants such as hormones and pesticides. From the perspective of insect protein production, however, these chemical pollutant risks can be addressed through targeted control of the feeding the insects—especially during the reproductive stage and biotransformation process to prevent or minimize the accumulation of heavy metals, toxins, veterinary drugs, and antinutrients from external environments (Cappelli et al., 2020).

Therefore, at present, the main limitation of the widespread use of insect proteins comes from the risk of allergies. The clinical manifestations of insect food allergy range from mild local reactions to severe systemic clinical manifestations. The reported symptoms can be divided into skin (e.g. urticaria, itching, rash, flushing, angioedema), gastrointestinal (e.g. abdominal pain, nausea, vomiting, diarrhea), and respiratory (e.g. asthma, dyspnea) (Pan et al., 2022). For example, a variety of potential allergens in silkworm pupae have been identified, such as 27-kDa glycoprotein, Bom m 9, thiol peroxiredoxin, chitinase and paramyosin, which can bind to IgE in serum from silkworm-allergic patients (He et al., 2020).

The current control measures include heating, microwave, glycosylation, high pressure, and enzymatic hydrolysis (Pan et al., 2022). However, it is critical to note that these interventions only reduce the allergenic potential of insect proteins; allergenic epitopes may persist even after processing, necessitating ongoing research into robust de-allergenization technologies and comprehensive allergen labeling protocols. Currently, the French company Ynsect is employing an integrated low-temperature enzymatic hydrolysis and high hydrostatic pressure (HHP) process to mitigate the allergenicity of yellow mealworm (*Tenebrio molitor*) protein. Specifically, low-temperature enzymatic hydrolysis involves using substrate-specific proteases to cleave peptide chains within allergenic proteins at controlled low temperatures (e.g., 4–30℃), thereby disrupting the structural integrity of IgE-binding epitopes. Concurrently, HHP applies isostatic pressures of 300–600 MPa for 5–10 min to induce non-covalent bond disruption (e.g., hydrogen bonds, hydrophobic interactions), leading to conformational changes in the secondary and tertiary structures of allergenic proteins. This dual-treatment approach not only reduces allergenic potential by altering antigenic surface features but also inactivates residual enzyme activity and eliminates microbial contaminants through pressure-induced cell membrane disruption (Sukan & Thunnop, 2024).

### Regulatory restrictions

With the development of the insect protein industry, countries are constantly introducing corresponding regulatory policies and standards. The EU mandates that novel foods should undergo a safety assessment by EFSA and obtain authorization from the European Commission; while whole insects have been exempted from prior regulatory frameworks pursuant to court rulings, they must still comply with transitional requirements. The United States implements a dual regulatory framework under FDA food regulations (GRAS or FAP systems) and USDA feed grading standards, with black soldier fly larvae already approved as aquatic feed by the USDA. China employs a directory management system for new food ingredients, requiring prior approval from the Ministry of Health. Japan grants exemptions for traditional insect foods under specific regulatory provisions. Canada and Australia adopt a case-by-case approval mechanism for novel food products, whereas Latin America and Africa predominantly rely on regional regulatory frameworks or traditional dietary practices (Gasco et al., 2020).

The growth of this industry is somewhat restricted due to outdated food and feed regulations covering insect use. For instance, Regulation (EU) 2015/2283 of the European Parliament and of the Council stipulates that whole insects and their derived products are classified as “novel foods”. Under this new regulation, insect food products may only be marketed when authorized after a safety assessment by the European Food Safety Authority (EFSA) (Sharma et al., 2024). This regulatory regulation promotes the production and sale of authorized insect-derived proteins but also means that insect-derived proteins not covered by the regulation no longer have the possibility of production. The application fee for EFSA’s new food certification is over 500,000 euros, and the approval cycle is about 2 years, which makes it difficult for small and medium-sized enterprises to afford.

In addition, as regulatory policies are gradually introduced across countries, a key issue is the lack of stable and consistent cross-border regulatory systems: for example, in Australia, house crickets, super mealworms, and mealworm beetles are not classified as novel foods (DiGiacomo et al., 2019); in Canada, novel foods definitions exclude traditional foods from other countries; while in the EU under its Novel Food Regulations and the US under GRAS/FAP rules, all insect-based foods require thorough safety assessments. Consequently, global insect companies must prepare different applications or notifications for the same product across national authorities due to varying requirements, which poses significant obstacles to developing the insect protein industry (Lhteenmki-Uutela et al., 2021). Regulation remains a key constraint on the growth of the insect protein industry. Notwithstanding significant progress, regulatory agencies must accelerate policy advancement and deepen their efforts to fully realize the industry’s potential.

## Conclusion and future perspectives

Advancements in biotechnology and production processes are unlocking unprecedented opportunities for insect proteins and peptides development to address the escalating global demand for sustainable protein sources. The nutritional profile of insect-derived proteins and peptides—featuring balanced amino acid compositions and bioactive peptides—fulfills premium requirements across human food, livestock feed, and pet nutrition sectors. Concurrent pharmaceutical research has identified insect-derived bioactive compounds with promising applications in immunomodulation, antimicrobial therapies, and oncology.

From a production perspective, characterized by rapid growth cycles, high fecundity, and low operational costs, insect cultivation provides an inherently scalable solution that aligns with projected protein demands. However, due to fragmentation in the insect protein processing chain and high investment thresholds in large-scale protein processing and extraction technologies, low consumer acceptance due to cultural biases and sensory perception, and fragmented global regulatory policies, the widespread application of insect protein still faces limitations. To address these challenges, food scientists in the industry are placing increasing emphasis on innovative extraction and processing technologies to enhance the functionality of insect protein products and reduce production costs. Meanwhile, there is active development of rapid and efficient quality control measures to ensure product safety. Collectively, these efforts are dedicated to the development of low-cost and efficient processing technologies and quality control measures suitable for large-scale production. In addition, integrating insect protein with daily food (e.g., hamburgers, sausages, pasta, and bread) can optimize sensory perception, improve consumer acceptance, and promote its application in mainstream foods.

From a policy perspective, the divergent cross-border regulatory frameworks, rooted in national regulatory disparities, represent the principal barrier to the globalization of the insect protein industry. This regulatory divergence forces multinational enterprises to incur substantial and repetitive compliance costs, thereby impeding international trade efficiency. Moreover, the prohibitive costs of regulatory certification further constrict the operational margins of small and medium-sized enterprises (SMEs). Furthermore, the delays in policy updates relative to technological innovation undermine industry competitiveness. It is imperative to expedite policy harmonization and establish a stable, unified cross-border regulatory system to unlock the industry’s full potential.

From a production perspective, stakeholders in the food industry should focus on the optimization of insect protein processing and extraction technologies to improve production efficiency and reduce costs. and strengthen cooperation with retailers, research institutions, and governments to build a stable industrial cycle to enhance resource utilization and risk resistance. At the market level, market segmentation can be based on regional culture. In traditional consumption areas such as Africa and Southeast Asia, the cultural embedding of insects (such as traditional recipes and medicinal values) can be emphasized, and localized products (such as fried locusts and silkworm pupae snacks) can be promoted; in non-traditional regions such as Europe and America, it is necessary to focus on the “invisible addition” strategy, such as incorporating insect powder into mainstream food forms such as hamburgers and protein powder, to avoid presenting the entire insect. In addition, targeted product development can be carried out for different consumer preferences, such as targeting health-oriented consumers, highlighting the nutritional advantages of insects (such as high protein, low fat, and rich in essential amino acids), developing fitness protein bars and dietary supplements; or using environmental narratives to attract sustainable consumer groups and promoting the environmental footprint of insect farming on product packaging (such as insect farming occupying only 10–40% of the land/water resources used in traditional animal husbandry). At the policy level, it is necessary to closely track policy developments in order to adjust production strategies in a timely manner.

Overall, insect proteins and peptides offer a revolutionary solution to address global protein shortages, thanks to their nutritional value, environmental sustainability, and multifunctional bioactivity. Although challenges such as poor processing efficiency, low consumer acceptance, and fragmented regulatory systems limit their large-scale application, the joint coordination and advancement of biotechnology, market strategies, and regulatory policies will transform insect-derived protein and peptide products into mainstream components of sustainable food, feed, and pharmaceuticals systems.
